# Synthesizing Liquid Fuels Over Carbon‐Based Catalysts Via CO_2_ Conversion

**DOI:** 10.1002/advs.202410280

**Published:** 2025-02-26

**Authors:** Cederick Cyril Amoo, Qingjie Ge, Vitaly Ordomsky, Jian Sun

**Affiliations:** ^1^ Dalian National Laboratory for Clean Energy Dalian Institute of Chemical Physics Chinese Academy of Sciences Dalian 116023 China; ^2^ University of Chinese Academy of Sciences Beijing 100049 China; ^3^ Université Lille Cité Scientifique Bâtiment C3 Villeneuve d'Ascq Cedex 59650 France

**Keywords:** carbon materials, CO_2_ conversion, green chemistry, sustainable liquid fuels

## Abstract

The unique characteristics of carbon materials make them flexible for applications in heterogeneous catalysis. Their interest is expanding in the conscious efforts being made toward sustainable fuel production. A notable application is the heterogenous conversion of CO_2_ to liquid fuels, which exploits the characteristics of carbon materials, taking advantage of their electronic configurations, high surface area, pore properties, and synergistic role in catalysis. In this review, a critical overview of this rapidly developing field is presented. Various carbon allotropes and derivatives, as well as some strategies for fabricating carbon‐based catalysts are keenly highlighted within thermal‐, electro‐, and photocatalytic CO_2_ conversion to liquid fuels. Distinct emphasis is placed on the role of different carbon materials by investigating the unique synergy attained at catalyst interfaces, the physicochemical properties attained, and their influence in enhancing the specific liquid fuels synthesis. Finally, the work is concluded, followed by an outlook detailing key challenges that need addressing.

## Graphical abstract

Carbon‐for‐Carbon‐Transformation of CO_2_ to liquid fuels can sustain the energy industry, reducing concerns associated with recent climate changes. Carbon‐based catalysts make a good choice on accessibility and cost constraints, aside from versatile characteristics. This work reports the growing application of carbon catalysts, outlining the progress, prospects, and challenges in fuel synthesis from CO_2_.

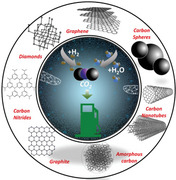

## 1 Introduction

Carbon materials have been essential across various chemical industries, which includes the catalysis sector. Their versatility to exist in different forms has made them attractive for various catalytic applications.^[^ ^1^, ^2^, ^3^ ^]^ Various forms such as graphene, fullerenes, carbon nanotubes, and bulk carbonaceous materials such as activated carbon are well recognized in the catalysis sector.^[^ ^4^, ^5^, ^6^ ^]^ Generally, carbon materials perform the functions of a support for metallic catalysts.^[^ ^7^, ^8^ ^]^ However, some recent applications use carbon as catalysts, with other elements in the absence of any metallic species.^[^ ^9^, ^10^, ^11^ ^]^ Thus, the use of carbon materials as catalysts keeps expanding and has found increasing application in the growing catalytic conversion of CO_2_.

Achieving “**carbon neutrality**” or what is now popularly called a “**net zero**” is a target to realize across many sectors of this current industrial regime.^[^ ^12^, ^13^, ^14^ ^]^ Today's increasing energy demand has a huge toll on the finite “fossil fuels,” which also consequentially emit enormous amounts of greenhouse gases during exploitation and use. Considering available avenues, “salvaging waste” stands among the majority to emphatically reduce greenhouse gas emissions to ultimately ensure environmental safety.^[^ ^15^, ^16^, ^17^ ^]^ Ubiquitous greenhouse gases such as CO_2_ are most difficult to contain and manage; thus, it is essential to find sustainable ways to utilize them.^[^ ^18^, ^19^ ^]^ Currently, CO_2_ is being transformed back into carbon‐based fuels via a few catalytic processes.^[^ ^20^, ^21^, ^22^, ^23^, ^24^, ^25^, ^26^, ^27^, ^28^ ^]^ The past 20 years have seen enormous progress in this area with over 100 000 publications encompassing various CO_2_ utilization processes. The fuels produced from CO_2_ conversion are normally hydrocarbons and oxygenates. Deductions from literature suggest gaseous products are easily acquired; thus, the challenge mostly lies in the selective production of liquid fuels. The success in synthesizing these liquid fuels will highly be dependent on the nature and composition of a catalyst system. Conventionally, transition metal catalysts such as Fe, Co, Pt, Pd, Cu, Zr, Zn, and Ni are often chosen in fabricating catalysts for CO_2_ conversion reactions. However, due to limitations in the selective production of liquid products, materials such as Al_2_O_3_, SiO_2_, TiO_2_, zeolites, and carbon have been introduced as supports for the strategic catalyst designs which have enhanced the targeted product selectivity and improved overall catalyst activity.^[^ ^29^, ^30^, ^31^ ^]^ Among all various supports, carbon materials are of high interest as they possess versatility accompanying tunable characteristics.^[^ ^32^, ^33^, ^34^ ^]^

**Figure 1:**
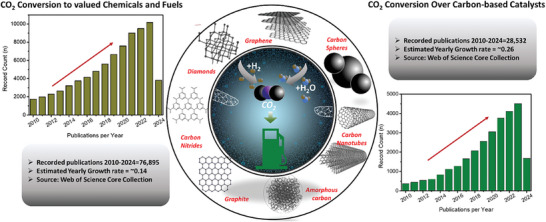
A simple scheme showing the status quo of CO_2_ conversion to chemicals and subsequent use of carbon materials in the various CO_2_ conversion processes (Data for the graphs were obtained from the Web of Science Core Collection with keywords presented in the appendix of this work).

Advances in CO_2_ valorization have been increasing rapidly over the years with a constant increase in technology commercialization and the number of publications. As shown in **Figure** **1**, over 76 000 catalytic CO_2_ conversion publications have been recorded in the past 13 years, out of which 37% were carbon‐based catalysts. Aside from expediency for their production and economic advantages over other supports, the characteristics of carbon materials can be tuned to enhance the dispersion of the metallic active phases and enhance the diffusion of reactants, intermediates, and products.^[^ ^35^, ^36^, ^37^ ^]^ The steric properties can be tailored to assume certain configurations that can facilitate specific product selectivity.^[^ ^38^, ^39^, ^40^ ^]^ More importantly, carbon‐supported catalysts can replace traditional metal oxide supports, which are challenged with stronger metal support interactions by weakening the interaction to improve the reducibility of metallic phases, ultimately enhancing the catalytic activity. Recent reviews have identified the role of carbon in carbon‐supported catalysis of the CO_2_ conversion reactions. These works have generally identified carbon as being tailor‐possible, hydrophobic, resistant to acid‐based media, and affordable over other conventional supports. Popular carbon‐supports such as carbon nanotubes (CNT's),^[^ ^41^, ^42^, ^43^ ^]^ activated carbon (AC),^[^ ^44^, ^45^ ^]^ carbon spheres, graphite, and carbon black have positively influenced the success of liquid fuels synthesis via electro‐,^[^ ^46^ ^]^ photo‐,^[^ ^47^, ^48^ ^]^ and thermocatalytic processes^[^ ^48^ ^]^ of the CO_2_ conversion reactions. All these various reviews have been specific in focus, either being the reaction type^[^ ^49^, ^50^, ^51^, ^52^, ^53^, ^54^ ^]^ or carbon type.^[^ ^51^, ^52^, ^53^, ^55^, ^56^ ^]^ To this day, there is no complete compiled document that has detailed the influence of carbon characteristics in CO_2_ conversion to liquid fuels across the various reaction types. This has limited the knowledge of any prospective use of carbon materials for major advancements across the different processes.

In this review, details on various characteristics of carbon materials exploited in the catalysis of CO_2_ conversion reactions are discussed, emphasizing their influence in liquid fuels synthesis over electro‐, photo, and thermocatalytic processes. Chronologically, the brief introduction detailing the background, purpose, and common carbon materials is highlighted in Chapter 1. Chapters 2, 3, and 4 detail some applications and characteristic influences of carbon in CO_2_ conversion to liquid fuels over thermocatalytic, electrocatalytic, and photocatalytic reactions, respectively. Detailed emphasis is placed on the physicochemical properties, synergy existing between the catalyst components, carbon type topologies, confinement effects, and overall catalyst configuration. More importantly, the operando mechanisms and kinetics accompanying the catalyst activity to liquid fuel synthesis biased toward the carbon influence within each reaction process are detailed. The work is concluded with a summary of the findings and outlook for further research.

### 1.1 Common Carbon Supports Used in Catalysis

Various forms of carbon can be recognized as catalytic materials with characteristics exploited for different catalytic processes. In this section, a few renowned carbon materials recognized as catalysts in converting CO_2_ are briefly described, with emphasis on their production and characteristics. Additionally, the **Table** **1** summarizes the properties of the common carbonaceous materials employed in fuel synthesis directly from CO_2_.

**Table 1:** Properties of some carbon materials in CO_2_ hydrogenation.

Carbon materials	S [m^2^ g^−1^]^a)^	V [cm^3^ g^−1^]^b)^	D [g cm^−3^]^c)^	Melting point [°C]
Activated carbon	1000–3500	0.6–2	1.8–2.1	3652–3697
Carbon nanotubes	120–500	2–2.5	1.7–2.1	3652–3697
Carbon fibers	1000–3000	0.3–0.7	1.75–2.0	3652–3697
Graphene	1500–2500	2–3.5	2.267	>3650
Graphite	10–100	0.01–1	2.266	>3600
Carbon black	50–250	0.2–2.5	0.09–0.34	3652–3697
Nano‐diamond	300–450	3.0–5.0	3.40–3.60	>4027
Graphitic nitrides	10–600	0.15–0.7	3.40–3.80	600–2950

^a)^ S‐Surface area;

^b)^ Pore volume;

^c)^ Density;

Note: Properties of the materials were obtained from CAS nos. of the commercial products.

#### 1.1.1 Activated Carbon (AC)

**Figure 2:**
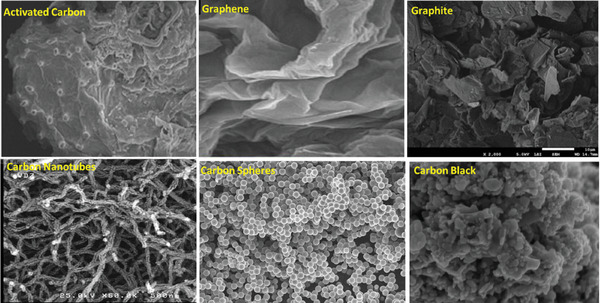
SEM micrographs showing the morphology of the ordinary forms of the carbonaceous supports used in CO_2_ conversion. Reproduced with permission.^[^ ^67^, ^68^, ^69^, ^70^, ^71^, ^72^ ^]^

Activated carbon, also known as activated charcoal, defines a group of carbon materials possessing a large surface area and porosity with the basic ability to adsorb chemicals from fluids. The adsorption ability is basically due to the strong van der Waals force which enables efficiency of the material in its applications. It is normally prepared by thermally treating the carbon‐based raw materials under anaerobic conditions at temperatures ranging between 673 and 1123 K.^[^ ^57^ ^]^ The resulting materials called char can be activated physically by treating it with steam, CO_2,_ or a mixture of both. The treatment eliminates debris and increases porosity and accessible phases of the formed carbon. The treatment can also be performed chemically by performing the thermal decomposition in the presence of H_3_PO_4_, H_2_SO_4_, HNO_3_, NaOH, KOH, or ZnCl_2_. This combines carbonization and activation in one step. The characteristic large surface area, porosity, and adsorption potentials have made them attractive for catalytic applications.^[^ ^58^ ^]^ AC can be recognized as the main support in redox reactions. **Figure** **2** represents a typical SEM image of AC derived from an organic source. The image confirms the amorphous nature of AC.

#### 1.1.2 Graphene and Graphite

Graphene is considered a new generation of carbon materials because of its unique physical and chemical characteristics. It possesses a 2D structure composed of a single carbon atom arranged in a honeycomb lattice. Graphite on the other hand has a 3D carbon structure with the carbon atoms sp^2^ hybridized. These materials have functioned well in recent catalytic applications due to their strong hydrophobicity and eased chemical functionalization, besides the common large surface area and pore characteristics. Graphite is normally prepared by high thermal treatment of carbon‐based materials, by carbon vapor deposition (CVD) from hydrocarbons, or thermal decomposition of unstable carbides.^[^ ^59^ ^]^ The resulting carbide demonstrates high thermal and mechanical stability and exceptional chemical resistance. Graphene can also be obtained from CVD on substrates or derived from graphite through exfoliation methods.^[^ ^60^ ^]^ These materials can be well‐recognized in recent catalytic hydrogenation applications.

#### 1.1.3 Carbon Nanotubes (CNT)

Carbon nanotubes (CNTs) are allotropes of carbon consisting of layer(s) of graphene. They currently represent one of the valued classes of nanomaterials with fascinating characteristics for applications in energy storage, gas storage, polymer reinforcements, and catalysis. Recognized methods for their production include arc discharge, chemical vapor deposition, flame synthesis, pyrolysis, and electrolysis. CNTs are classified as single‐walled carbon nanotubes (SWNTs) and multiwalled carbon nanotubes (MWNTs). SWNTs can be visualized using a tube length of graphene wrapped in the form of a cylinder. The circumference of the graphene sheet wrap should map onto one of the Bravais lattice vectors of the graphene plane sheet. The lattice vector is defined by two‐unit vectors (a_1_, a_2_) and a pair of integers (n, m). The MWNTs consist of a stack of graphene layers rolled up. CNTs have found applications as reaction interfaces, as catalysts, and catalyst support.

#### 1.1.4 Carbon Nitride (g‐C_3_N_4_)

Carbon nitride is a polymeric graphitic carbon material, g‐C_3_N_4_, consisting of C, N, and some impurity H, connected via tris‐triazine‐based patterns. Compared with other major carbon forms, it has electron‐rich properties, basic surface functionalities, and H‐bonding motifs due to the presence of N and H atoms. The common method reported for the synthesis of g‐C_3_N_4_ is the thermal treatment of various organic precursors. The most common precursors used for the chemical synthesis of g‐C_3_N_4_ are reactive nitrogen‐rich and oxygen‐free compounds containing pre‐bonded C─N core structures, such as triazine and heptazine derivatives.^[^ ^61^ ^]^ Antonietti et al. employed thermal polycondensation of cyanamide or dicyandiamide into g‐C_3_N_4_ under N_2_ flow.^[^ ^62^ ^]^ Zou et al. directly heated melamine to g‐C_3_N_4_ in a semi‐closed system.^[^ ^63^ ^]^ The large‐scale synthesis of g‐C_3_N_4_ has also been reported via the pyrolysis of urea. Carbon nitride continues to see increasing applications in photocatalysis, air purification, chemical transformation, and other important chemical sectors since its commencement.

#### 1.1.5 Carbon Spheres (CS)

Carbon spheres are defined as bulk spherical carbonaceous materials with characteristics of surface and pore properties, making them attractive materials for adsorption and catalysis. Carbon spheres can easily be obtained from various carbon sources; however, the use of bio‐resources makes their production less expensive and sustainable. Conventionally, carbon spheres are produced from the hydrothermal carbonization of the carbon source. The spherical final product comprises bulk carbon materials with functional surface groups. In catalysis, carbon spheres can be recognized in bio‐catalytic applications, redox applications, and hydrogenation reactions as catalysts or support. The functional surface groups have been well‐exploited in hydrogenation.

#### 1.1.6 Carbon Black (CB)

Carbon black is an important carbon support consisting of a combination of amorphous and quasi‐graphitic carbon materials. It is normally formed as a result of incomplete combustion of heavy petroleum and bio‐resources through natural and artificial activities. The microporous structure, high catalytic active surface area, high absorption capability, exceptional chemical stability, high conductivity, and low‐cost synthesis procedure make these supports suitable for use in large‐scale applications.^[^ ^64^ ^]^ In addition, there are a lot of particular oxygenated functional groups on the surface of CB that have been exploited across a few hydrogenation processes.^[^ ^65^, ^66^ ^]^

#### 1.1.7 Carbon Nanofibers (CNF)

Carbon nanofibers can be conceived as a matrix of graphitic carbon layers, as displayed in Figure 2. They possess a high thermal and electrical conductivity for various catalytic applications. They can well be recognized in hydrogenation reactions. It is reported that the CNF, like other carbon‐based materials, can exhibit unique surface properties that can improve their activity in hydrogenation reactions. CNFs are normally prepared by CVD but can also be obtained via hot filament‐assisted sputtering and template‐assisted methods.

## 2 Thermo‐Catalytic Conversion of CO_2_ to Liquid Fuels

**Figure 3:**
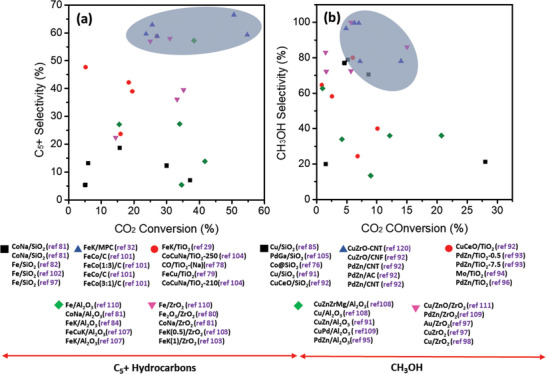
Recently reported activity of some popular supported catalysts in CO_2_ conversion to liquid fuels and accompanying data, highlighting the developments in carbon‐based catalysts.

CO_2_ can be converted to liquid fuels over catalysts in a thermally controlled reaction. Oxygenates and heavy hydrocarbons such as CH_3_OH, DME, gasoline, and EtOH have been successfully produced from the conversion of CO_2_. Major influencers are the reaction parameters and nature of catalysts. While the temperature and pressure are key in mainly influencing the product distribution, the nature of the catalyst can additionally determine the reaction path. The concept of CO_2_ conversions employs the reduction of CO_2_, mostly with H_2_ to reactive C1 species, which proceed to subsequent chain growth or hydrogenation reactions. Conventionally, supported or promoted oxide‐based metal catalysts are chosen for the selective production of liquid fuels. Zeolites in particular have been favorites for the selective production of liquid hydrocarbons, DME, and also some alcohols due to their hydroprocessing ability.^[^ ^73^, ^74^ ^]^ Nevertheless, carbon materials remain attractive for the reasons identified earlier above. Carbon materials used in the thermal catalytic CO_2_ hydrogenation reactions have augmented the metallic species as support to form a unit composite. The support‐comparison presented in **Figure** **3** showing the activity of different liquid fuels confirms the remarkable performance of carbon‐supported catalysts (over other oxide‐based supports) in both CO_2_ conversion activity and liquid fuel selectivity. Cumulative deductions indicate that the bulk carbon well anchors the metallic species and ensures the high dispersion of the metal particles, a key characteristic to improving catalyst performance.^[^ ^75^ ^]^ The carbon normally has a low metal support interaction with the metallic species, inhibiting the formation of nonreducible metal‐support complexes that follow other oxide‐based supports. In addition, reports are that the carbon can facilitate the formation of phases that favor selective production of certain long‐chain hydrocarbons and oxygenates.^[^ ^76^ ^]^ Furthermore, surface functional groups exist on the carbonaceous material and can be advantageous to influence product distribution if well‐tailored.^[^ ^77^, ^78^ ^]^ The thermocatalytic CO_2_ conversion is the most advanced for a variety of liquid fuel synthesis showing promise with an already industrial recognition for most liquid fuel types. With sustainability at the forefront, detailing the role of carbon as identified in recent literature will be essential to propel the studies on carbon‐based catalysts in CO_2_ conversion to liquid fuels.^[^ ^79^, ^81^, ^82^, ^83^, ^84^, ^85^, ^86^, ^87^, ^88^, ^89^, ^90^, ^91^, ^92^, ^93^, ^94^, ^95^, ^96^, ^97^, ^98^, ^99^, ^100^, ^101^, ^102^, ^103^, ^105^, ^106^, ^107^, ^108^, ^109^, ^110^, ^111^, ^112^, ^113^, ^114^ ^]^

### 2.1 CO_2_ to Oxygenates

The direct conversion of CO_2_ to oxygenates normally follows a hydrogenation path in the thermocatalytic approach; although, some reactants other than H_2_ are also being used in the conversion process over specific catalysts.^[^ ^115^ ^]^ A group of metal‐based catalysts has shown promise for alcohol production via CO_2_ conversion. While various supports can also be recognized within this process, desired characteristics that favor the production of alcohols with limited side reactions determine the choice of support. With this reference, some carbon‐based materials have shown promise after being investigated as a catalytic material in the synthesis of oxygenates from CO_2_. Besides anchorage, other desirable physical and chemical properties of carbon have been beneficial within this process.

#### 2.1.1 Effects of Physical Properties

**Figure 4:**
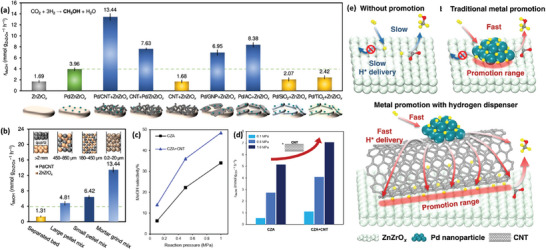
Physical effects of carbon materials represented in a) the influence of carbon on methanol formation rates and b) the influence of proximity modes. e) The schematic shows the adsorption and delivery of “H^+^” for enhanced CH_3_OH production. c,d) Influence of carbon of MeOH formation rates on conventional CZA catalysts. Reproduced with permission.^[^ ^126^ ^]^ Copyright 2023, Springer Nature.

A catalytic system comprising Pd–ZnO supported on MWCNTs for selective oriented hydrogenation of CO_2_ to methanol was reported.^[^ ^116^ ^]^ MWCNTs perform the dual function of metal anchorage and reaction promoter. The surface concentration of the Pd particles increased with the introduction of MWCNTs to the reaction, which was the catalytically active site for methanol generation. The turnover frequency for CO_2_ hydrogenation using MWCNTs as catalyst was 1.17 and 1.18 times superior to activated carbon (AC) and ɤ‐Al_2_O_3_.^[^ ^117^ ^]^ In a work by Deerattrul et al.,^[^ ^117^ ^]^ a high surface area carbon material was synthesized to support a “CuZn” composite in CO_2_ hydrogenation to CH_3_OH. The high activity realized was attributed to the high surface area of meso‐carbon which suppresses metal agglomeration and enhances CO_2_ adsorption. Other forms of carbon such as activated carbon (AC), CNTs, and rGO have all recorded applications for the synthesis of CH_3_OH from CO_2_.^[^ ^118^, ^119^, ^120^, ^121^ ^]^ MOF‐derived Co/C–N has been reported by Lian et al. for the synthesis of CH_3_OH in CO_2_ hydrogenation. It was observed that the lower calcination temperature, as in Co/C–N‐600, resulted in smaller metallic Co particles and a much higher catalyst surface area which was key to improving CH_3_OH selectivity.^[^ ^122^ ^]^ In all these works, the carbon had functioned well more for dispersion, improving surface metallic species and enhancing CO_2_ adsorption with an eventual improvement in CH_3_OH synthesis. Carbon‐based catalysts have also been used to realize higher alcohols directly in the CO_2_ hydrogenation. A recent work by Wang et al. realized a 35.0% selectivity for C_2_+ alcohols. The in‐depth characterizations and calculation revealed that the interfaces, proximity modes of the multifunctional catalysts, as well as the intermediates, prominent being aldehyde species, played a vital role in enhancing the overall CO_2_ hydrogenation to ethanol.^[^ ^123^ ^]^ A series of supported Mo–Co–Ks was investigated in CO_2_ conversion to different products. It was observed that the C_2_+ alcohols’ selectivity was high over the AC‐supported catalysts. Interpretations from the various characterizations suggest that the carbon material could anchor more Mo species on its surfaces, with a generally high adsorption capacity concluded to have resulted in the high C_2_+ alcohol selectivity.^[^ ^124^ ^]^ Din et al. synthesized a wide range of carbon nanofibers supported bimetallic catalyst–copper oxide/ zirconia (CuO/ZrO_2_/CNF) for the hydrogenation of CO_2_ to methanol.^[^ ^125^ ^]^ These catalysts were prepared via the deposition precipitation method with varying CuO weight percentages (5 wt%, 10 wt%, 15 wt%, 20 wt%, and 25 wt%). Upon increase in CuO loading above 15 wt%, agglomeration takes place within the particles, which in turn reduces the catalyst surface area, resulting in lower methanol yield. A linear relationship in the surface‐modified carbonaceous nanomaterials for CO_2_ hydrogenation was found between the Cu surface area and methanol yield. An increase in surface area can provide more atomic hydrogen, which is supplied to ZrO_2_ basic sites for CO_2_ reduction, which results in higher methanol yield. A much more detailed investigation has been realized for dispensing hydrogen to active ZrZnO_2_ surface by Lee et al., as shown in the **Figure** **4**.^[^ ^126^ ^]^ Introduction of carbon improves the MeOH formation rate by increasing the H_2_ adsorption and delivery to the ZrZnO_2_ surfaces with a large promotion range. This was observed for every carbon‐based catalyst over other supports, with the Pd/CNT + ZrZnO_2_ catalyst demonstrating the highest MeOH formation rate. The effects of Pd/CNT and ZrZnO_2_ proximity were further investigated. The mortar grind mix option demonstrated highest formation of MeOH, indicating activity was not determined by gas phase transport but rather by the high proximity with the carbon interface. The direct influence of the carbon on MeOH generation was further confirmed with a commercial CZA mixed with CNT (Figure 4c,d).

#### 2.1.2 Effects of Chemical Properties

**Figure 5:**
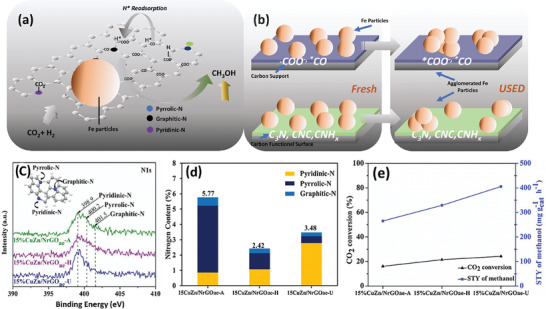
Functionalized carbon supported metal catalysts showing a) the adsorption sites for CO_2_, H* reabsorption sites, with different nitrogen functional groups. b) The influence of carbon surface functionality on metal particle size, fresh, and after activity; and c,d,e) influence of nitrogen functional groups on CO_2_ hydrogenation activity. Reproduced with permission.^[^ ^132^ ^]^ Copyright 2019, Elsevier.

Carbon materials can enhance the CO_2_ conversion process through the interactions with the catalyst's surface. Normally these can take the form of aiding the formation of catalytically active species such as carbides or partaking in the reaction process through direct interactions with the reactants and intermediates. Successful confinement of Mo species in graphitic carbon provided a platform for the easy formation of Mo_2_C.^[^ ^127^ ^]^ This phase created a heterojunction with MoSe_2_, and together with the in situ formed carbon phase, synergistically enhanced CO_2_ activation and MeOH production. Carbon surfaces can provide a platform for easing Fe_2_C_5_ formation, which is essential for the coupling of CH_2_* and CHO* to CH_2_CHO* for alcohol formation. However, other functional groups can be induced using metals, heteroatoms, and acid functionalization to change the surface chemistry for enhanced activity. A “Mn,” “Cu,” and “K,”‐ modified Fe_5_C_2_ catalyst improved the selectivity for C_2_+ alcohols with significant proportions of the products being propanol and butanol.^[^ ^128^ ^]^ The unpromoted iron carbide has a strong ability for hydrocarbon chain growth, leading to the fast formation of HCs rather than HAs. The introduction of the other metals can equally modify and influence the performance of the iron carbide catalyst. The presence of ‘K’ modification in this particular catalyst increased the CO_2_ adsorption and suppressed the deep hydrogenation of carbon during the reaction, which oriented products more toward alcohols.^[^ ^129^ ^]^ Zha et al.^[^ ^129^ ^]^ reported that the surface of CNT could be functionalized and exploited to improve the activity and enhance DME yield. The acid functionalization of the carbon led to the introduction of more C‐functional groups and also to the reduction of the hydrophobic properties of the carbon. This was reported to improve the reversible reabsorption of hydrogen (**Figure** **5**a). This characteristic high H_2_ adsorption of carbon had been reported by several and tipped as a reference for improved alcohol selectivity in CO_2_ hydrogenation.^[^ ^126^ ^]^ The surfaces of carbon can also contain oxygen vacancies reported as necessary for CH_3_OH synthesis from CO_2_ hydrogenation. Nitrogen is also a major functional material on carbon in CO_2_ hydrogenation reactions. They can exist as pyridinic, pyrrolic, and quaternary N‐groups on carbon. They perform the general role of enhancing metal reduction, improving surface basicity, and enhancing CO_2_ conversion.^[^ ^33^, ^130^, ^131^ ^]^ A study on the effects of different nitrogen functional groups on carbon has revealed that the pyridinic‐N performs better for CH_3_OH selectivity and CO_2_ conversion.^[^ ^132^ ^]^ Results from the characterizations indicate that the catalyst dominating in pyridinic‐N has a lower reduction temperature, with the abundant surface N‐groups donating lone pairs of electrons for enhanced performance. More importantly, the conversion and MeOH selectivity are linearly correlated with the amount of pyridinic‐N present (Figure 5e). Other works such as that reported by Donphai et al. also conclude that functionalizing carbon materials with nitrogen species can supply lone pair electrons to form metal complexes for enhanced CH_3_OH formation.^[^ ^133^ ^]^ Wang et al. fabricated CNTs‐supported Cu/ZrO_2_ catalysts for hydrogenation of CO_2_ to methanol.^[^ ^134^ ^]^ The nitrogen groups introduced on the CNTs during the preparation increased the dispersion of CuO on the support surface and enhanced H_2_ and CO_2_ adsorption. In a thermally controlled reaction, the particle size of the metal plays a key role in defining the catalyst's performance. The supported metal catalyst controls this by providing anchorage regulating the particle size during the reaction. While a stronger interaction can be obtained over oxide‐based supports, keeping the metal particles more stable, the formation of unreducible oxides reduces the number of active phases. Recent studies have also shown the enhanced improvement of SMI over carbon materials, with highly dispersed active phases for methanol production.^[^ ^135^ ^]^ An important observation is the contribution of acidic oxygen functional groups, which results in the formation of slightly larger metallic particles; while, the basic N‐functional groups benefit the reduction of Cu crystal size for enhanced methanol selectivity in the final product. A schematic adaptation is presented in Figure 5b to reveal the influence of the surface functional groups on particle sizes. These traits keep carbon as a stable material with merits for catalyst development in CO_2_ hydrogenation to oxygenates. Moreover, it has been concluded from several studies that carbon supports normally outperform traditional oxide supports in CO_2_ hydrogenation to methanol.^[^ ^119^, ^129^ ^]^

### 2.2 CO_2_ to Heavy Hydrocarbons

From the literature, carbon materials have aided the production of some heavy hydrocarbons. When promoters are well configured with the Fe and carbon support, the C–C coupling ability increases.^[^ ^136^ ^]^ Generally, two major routes are considered for CO_2_ conversion to hydrocarbons. An oxygenate (normally methanol) mediated route (MTH) which normally employs solid acids for subsequent methanol conversion or a more direct route using promoted/supported metal‐based catalysts with CO as key intermediate (CO_2_‐FTS). Carbon materials have been well deployed in the production of methanol from CO_2_, which is a key intermediate in the MTH process as presented above. However, this section reveals progress in the use of carbon‐supported catalysts in CO_2_‐FTS for direct liquid fuel production.

#### 2.2.1 Effects of Physical Properties

A key characteristic following carbon catalysts in CO_2_ hydrogenation is the graphitization process. It has been reported that carbon with a high graphitization degree has a stronger electron transfer capability to promote CO activation, making it key to influencing the FTS step. A Fe–K/CNT catalysts’ catalyst was developed for the direct hydrogenation of CO_2_ to heavy hydrocarbons. The reaction optimized over a K/Fe ratio variation showed a C_5_+ selectivity of 56% with improved graphitization as a key characteristic for enhanced C_5_+ selectivity. However excessive graphitization of the carbon support caused by excessive loading of “K” slightly restricted C_5_+ formation.^[^ ^80^ ^]^ Another thorough investigation was done by Guo et al. using different carbon materials as support for CO_2_ hydrogenation.^[^ ^137^ ^]^ The carbon materials prepared to encapsulate “Fe” and “K” demonstrated high selectivity for C_5_+ hydrocarbons with graphene supports enhancing C_5_+ selectivity to 53%. The results indicate that the surfaces of the GA‐supported catalysts were well covered with carbon possessing graphitic components that suppressed the sintering of metallic catalysts. Another comparison involving CO_2_ hydrogenation activity over FeK/SWNTs against FeK/MWNTs revealed that the SWNT had a large curvature with a stronger binding to lighter olefins that enhanced the selectivity for C_5_+ olefins over FeK/MWNTs.^[^ ^138^ ^]^ The conclusions from this work reveal the potential of carbon to facilitate heavy hydrocarbon formation by increasing the retention of lighter carbon species for longer chain growth within the pores of CNTs. The selectivity for C_5_+ hydrocarbons is also improved by employing mesoporous carbon supporting Fe in CO_2_ hydrogenation. The unique pore geometry of the meso–carbon ensures rapid diffusion and can accommodate large molecular hydrocarbons.^[^ ^32^ ^]^ More recently, the effects of existing synergy between the metal and carbon interface have been investigated in CO_2_ hydrogenation.^[^ ^139^ ^]^ Revelations and conclusions indicate that a stronger intimacy between the metals and the carbon matrix is advantageous for C─C coupling in heavy hydrocarbon formation. This makes encapsulating metal particles within carbon the best choice in targeting longer‐chained hydrocarbons. The general influence of carbon was also observed to be positive in the hydrocarbon chain growth step, suggesting the key role of carbon.

**Figure 6:**
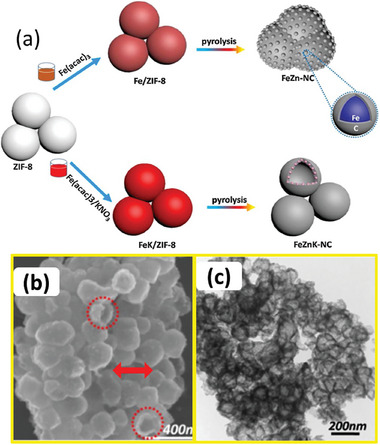
A MOF‐derived FeZnK‐NC catalyst showing the detailed stages of formation and final particle structures in b) SEM and d) TEM. Reproduced with permission.^[^ ^36^ ^]^ Copyright 2018, Elsevier.

Some MOF‐derived Fe─C catalysts have also been reported to improve the selectivity for liquid fuels. The pyrolysis of the MOFs normally results in the metal particles stacking within a carbon matrix. Fe‐MIL‐88B derived Fe–C by pyrolysis demonstrated improved selectivity for C_5_+ in CO_2_ hydrogenation.^[^ ^140^ ^]^ The revelations from the work indicate that MOF‐derived catalysts possess metal particles more dispersed within the carbon matrix that improve the chain growth ability and stable performance of the catalyst. In another instance where Fe@C composites were obtained from the pyrolysis of Fe/ZIF‐8 and FeK/ZIF‐8, the authors reported that carbon encapsulating the metal particles improved the stability of the overall catalyst.^[^ ^36^, ^141^ ^]^ These reveal the influence of carbon materials on liquid hydrocarbon production in CO_2_ hydrogenation. The interesting characteristic of the carbon‐based catalysts derived from the MOF structures, as demonstrated in **Figure** **6**, is the localization of the metal particles within the carbon matrix, giving a stable structure and anti‐sintering properties to the metals. More importantly, the stronger intimacy between the metal and carbon interface increases its propensity to improve heavy hydrocarbon selectivity.

#### 2.2.2 Effects of Chemical Properties

**Figure 7:**
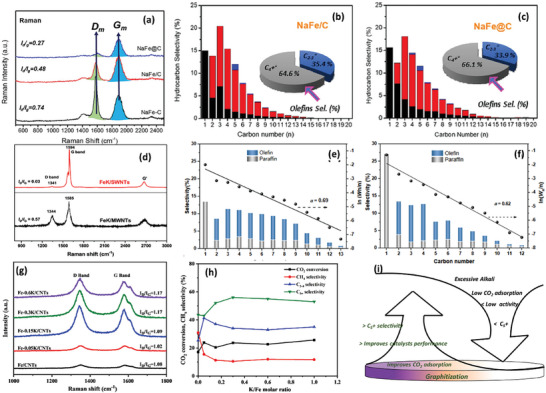
Raman spectroscopy and hydrocarbon product distribution showing the influence of graphitization on hydrocarbon chain growth. Reproduced with permission from ref. [139] (a‐c) 2023, Elsevier, ref. [138] (d‐f) 2020, American Chemical Society. and, ref. [80] (g,f) 2021, WILEY. (i) Summarized influence of graphitization and excessive alkali in CO_2_ hydrogenation.

Initially stated, the graphitic phases of the catalyst are major influencers for the CO_2_ conversion and hydrocarbon chain growth. Amoo et al.^[^ ^139^ ^]^ and Wang et al.^[^ ^138^ ^]^ directly demonstrated this fact in **Figure** **7**, with the low *I*_d_/*I*_g_ ratio catalysts showing results for higher heavy hydrocarbons and olefins in products. However, different metals on carbon phases demonstrate different degrees of graphitization. An investigation performed by Chen et al. using Fe/AC with a range of metal promoters as catalysts revealed that the different metals resulted in different *I*_d_/*I*_g_ ratios.^[^ ^142^ ^]^ They concluded that the catalysts showing a rapid deactivation rate tended to possess a large *I*_d_/*I*_g_ ratio, suggesting that the deposition of disordered carbon species on the active sites has a large impact on deactivation. Excess addition of alkali can also compromise the graphitization process, which can hamper CO transformation.^[^ ^143^, ^144^ ^]^ The Figure displays the activity comparison of *I*_d_/*I*_g_ ratios on catalyst activity. Clearly, a larger *I*_d_/*I*_g_ ratio improves the selectivity for C_5_+ hydrocarbons; while, high alkali content reduces the graphitization process.

In the instance where Fe@C composites were obtained from the pyrolysis of Fe/ZIF‐8 and FeK/ZIF‐8, the authors reported that carbon encapsulating the metal particles improved the stability of the overall catalyst.^[^ ^36^ ^]^ The pyrolysis process could also result in an early formation of carbide phases key for the reaction process. In addition, the “N” component was identified as an effective electron donor, key for CO_2_ dissociation and C─C coupling. A work reported by Fu et al.^[^ ^21^ ^]^ concluded that the oxygen functional surfaces of carbon could improve CO_2_ adsorption and suppress C_1_ products in the final product stream.^[^ ^145^ ^]^ Another key chemical contributor to the use of carbon as support is the enhanced H_2_ spillover as reported by Davide Mattia's group.^[^ ^145^, ^146^ ^]^ They concluded that the bridge or a strong interaction forming between the metal and carbon surfaces has the propensity to increase surface “H” atoms for improved reduction of CO_2_. Besides, a general conclusion by Wang et al. indicated that carbon materials tend to have a high hydrogen spillover capacity than conventional silica oxides.^[^ ^147^ ^]^ **Table** **2** accumulates recent literature on carbon‐supported catalysts in CO_2_ hydrogenation to C_5_+ hydrocarbons. High selectivity reaching 63% with yield reaching over 50% in products makes carbon materials promising for catalyst fabrication in CO_2_ hydrogenation.

**Table 2:** Comparison of the results in literature works for CO_2_ hydrogenation to C_5+_ hydrocarbons over carbon‐based catalysts.

Catalysts	Reaction conditions		CO_2_ conv. [%]	CO sel. [%]	Hydrocarbon distribution [%]			C_5+_ yield [%]	Ref
	T/P/GHSV^a)^	H_2_/CO_2_			CH_4_	C_2_–C_4_	C_5+_		
Na–CoFe_2_O_4_/CNT	340/1/3600	3	34.4	18.6	14.7	44.4	40.9	11.5	[148]
KFe/AC	300/2.5/2000	3	41.0	6.5	18.0	42.0	40.0	15.3	[142]
FeZn‐NC	320/3/7200	3	29.3	19.9	20.7	45.1	34.2	8.0	[36]
K/Fe–C	320/2/4800	2.5	35.6	20.9	15.3	43.9	40.8	11.5	[149]
Fe/C–K@NC‐350/600	340/3/5000	3	25.5	58.5	16.2	28.4	55.4	5.9	[150]
Fe–K/HPCMs‐1	400/3/3600	3	33.4	38.9	13.5	29.5	18.1	6.1	[35]
Fe/C–K	320/1/2240	3	28.0	22.6	24.0	38.1	37.9	8.2	[151]
N–K‐600‐0	400/3/3600	3	43.1	26.1	35.5	43.7	20.8	6.6	[140]
4K/Fe–C	300/3/2000	3	32.4	5.1	12.8	32.4	54.8	16.8	[141]
FeCo/C(1:1)	240/3/500	3	27.1	22.9	9.2	7.6	58.8	23.8	[104]
FeCo/C(3:1)	320/3/500	3	54.7	6.9	16.7	17.1	59.3	50.4	[104]
FeCo/C(3:1)	240/3/500	3	25.6	27.1	4.9	5.1	62.9	25.0	[104]
FeCo/C(1:3)	240/3/500	3	22.6	33.5	2.2	4.7	59.6	20.9	[104]
K–Fe/CNT	340/2/1200	3	35.0	12.0	26	46.1	30.0	N/A	[152]
Fe/C‐Bio	320/1/2400	1	31	23.2	11.8	38.1	63.8	N/A	[151]
NaFe@C	300/3/4000	3	38.6	6.0	13.1	38.1	48.8	N/A	[139]
FeK/MPC	300/2.5/8000	3	37.2	8.4	18.3	33.2	40.2	N/A	[32]
FeK/SWNTs	340/2/9000	2	52.7	9.6	13.5	31.1	55.4	N/A	[138]

^a)^ Notes: T/P/GHSV: Temperature [°C]/Pressure [MPa]/GHSV [mL·g_cat_^−1^·h^−1^].

## 3 Electrocatalytic CO_2_ Reduction to Liquid Fuels

CO_2_ can be converted to liquid fuels under electrocatalytic conditions. Electrocatalysis defines a reaction system occurring at an electrode–electrolyte interface, where the electrode plays the dual role of electron donor/acceptor. Under electrochemical conditions, a high overpotential is required, especially for CO_2_ reduction, in addition to challenges from low efficiencies and stability of the catalyst. Three main steps are recognized in a simple electrocatalytic reduction. First, the adsorption of the molecule on the surface of the electrocatalyst, electron transfer for reduction and reformation of bonds, and the final products formed are dissolved in the electrolyte. The formation of final products such as CH_3_OH and C_2_H_5_OH requires the transfer of more electrons; as such, an efficient catalyst is required to lower the energy barrier for selective production. In addition, the reaction competes with the HER; thus, materials capable of suppressing this side reaction are required.

Conventionally, transitional metals, conducting polymers, ionic liquids, and enzymes can be used as electrocatalysts for CO_2_ reduction. A problem confronting the use of metals as catalysts is the scaling relations among the adsorption energies of different intermediates, as rationalized by the d‐band model.^[^ ^153^, ^154^ ^]^ There are also limitations associated with high cost and competing excessive HER. A feasibility study done on potential industrial catalysts reveals carbon‐based, SACs, copper‐based, and molecular catalysts to be viable options. Carbon material has been abundantly employed within this domain and is recognized as a prospective material for catalysis on both small and larger scales. Carbon is normally used together with metals and in a unit composite catalyst or under metal‐free conditions with heteroatoms to replace metal‐based electrodes in CO_2_RR reduction reactions. It is also common to find carbon materials functioning as electrodes.^[^ ^155^, ^156^ ^]^ Common types are the glassy carbon and graphene. Glassy carbon has the advantages of low cost and low electrical resistivity, making it attractive for electrocatalytic reactions. Recent applications of glassy carbon electrodes have modified the structures with functional groups and other complexes, as well as other carbon‐based solids.

Some advantages of using carbon materials as catalysts include tailorable porous structures, high surface area for adsorption, and high electrical conductivity. Naturally, an unadulterated carbon material will display limited electrochemical activity; however, modifications in terms of hetero‐atom doping and metals can improve the electron density and the adsorption of CO_2_. Details of different carbon structures and unit composites developed to produce liquid fuels are discussed below.

### 3.1 Heteroatom‐Doped Catalysts

**Figure 8:**
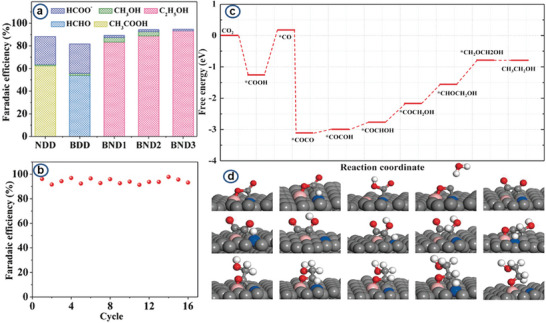
a) Faradaic efficiencies for CO_2_ reduction on NDD, BDD, BND1, BND2, and BND3 at −1.0 V. b) Faradaic efficiencies for CH_3_CH_2_OH during 16 consecutive runs for CO_2_ reduction on BND3 at −1.0 V. c) Free energy diagrams for CO_2_ reduction on (111) facet of BND. d) Energetically favorable structures for elementary steps of CO_2_ reduction on (111) facet of BND (gray = C, pink = B, blue = N, red = O, and white = H). Reproduced with permission.^[^ ^160^ ^]^ Copyright 2017, Wiley.

Heteroatoms including N (Nitrogen), B (Boron), and P(Phosphorous) are common to modify the carbon materials. A nitrogen‐doped mesoporous carbon material was investigated for the synthesis of ethanol via the CO_2_‐RR reaction.^[^ ^157^ ^]^ The mesoporous carbon was beneficial for the electrocatalysis process with large surface area and pores, chemical stability, and high electric conductivity. The “N” sites resulted in high electron density, which was crucial for CO dimerization toward C_2_H_5_OH formation. The faradaic efficiency for C_2_H_5_OH reached 77% at 0.56 V with an overpotential of 0.63 V. As stated earlier, nitrogen on carbon can exist in four forms being, pyridinic, pyrrolic, quaternary, and pyridine‐N‐oxide. Unlike under thermocatalytic conditions, the pyrrolic are more active in CO_2_RR with reduced activity to HER over the other forms.^[^ ^158^, ^159^ ^]^ While the uni‐heteroatom doped demonstrates improved selectivity to valuable carbon products, incorporating more than one heteroatom can demonstrate even better performance. This instance has been demonstrated by Liu et al.^[^ ^160^ ^]^ in a report with “B” and “N” co‐doped on diamonds for the formation of C_2_H_5_OH (**Figure** **8** and **Table** **3**). The results indicate that the co‐doped B and N catalyst has a more negative hydrogen evolution potential than the uni‐heterodoped carbon material. The faradaic efficiency for C_2_H_5_OH was 93.2%. Experimental analysis, coupled with DFT calculation, indicated the high activity and selectivity of BND for C_2_H_5_OH production mainly originated from the synergetic effect of “B” and “N” cooping and fine balance between “N” content and H_2_ evolution potential.

**Table 3:** Carbon‐based CO_2_ RR electrocatalysts for liquid fuels production.

Catalyst	Electrolyte	Current density [mA.cm^−2^]	Potential [V]	FE [%]	Products	Ref.
CuZn*x*/NGN	0.1 m KHCO_3_	4.0	−0.8	34.2	C_2_H_5_OH,	[166]
CuZn*x*/NGN	0.1 m KHCO_3_	4.0	−0.8	12.4	C_3_H_7_OH	[166]
GN/ZnO/Cu_2_O	0.5 m KHCO_3_	0.8	0.9	30.0	C_3_H_7_OH	[174]
N‐functionalized GO	0.1 m KHCO_3_	0.7	−0.4	36.4	C_2_H_5_OH,	[175]
PGA	0.5 m KHCO_3_	4.7	−0.8	48.7	C_2_H_5_OH,	[176]
BND	0.1 m NaHCO_3_	N/A	−1.0	93.2	C_2_H_5_OH,	[160]
C‐NC	0.1 m KHCO_3_	N/A	−0.56	77	C_2_H_5_OH,	[157]
MNCs	0.1 m KHCO_3_	N/A	−0.56	78	C_2_H_5_OH,	[177]
OD Cu‐C	0.1 m KHCO_3_	N/A	−0.5	34.8	C_2_H_5_OH,	[163]
MC‐CNT/Co	0.5 m KHCO_3_	N/A	−0.32	60.1	C_2_H_5_OH,	[178]

### 3.2 Metal‐Incorporated Carbon Catalysts

**Figure 9:**
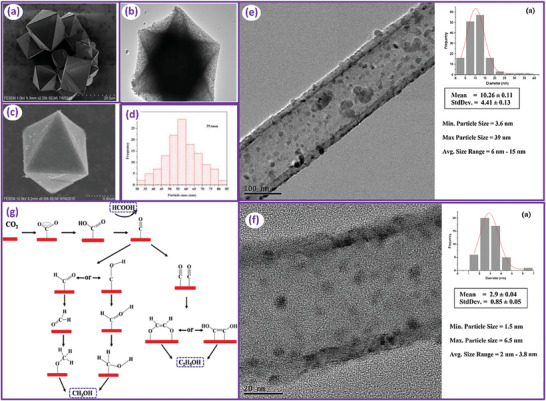
a–d) SEM, TEM, and particle size distribution of Cu on Cu‐HKUST derived Cu/C catalyst. Reproduced with permission.^[^ ^163^ ^]^ Copyright 2017, American Chemical Society. CNT supported Cu catalysts with particle size distribution prepared by e) impregnation and f) Cu nanowire deposition, Reproduced with permission.^[^ ^164^ ^]^ Copyright 2017, Elsevier. g) The proposed reaction scheme for alcohol formation from Cu/C catalysts.^[^ ^163^ ^]^

Some metal‐supported carbon catalysts have been used in the synthesis of some liquid fuels in CO_2_RR.^[^ ^161^, ^162^ ^]^ Among them, Cu‐based catalysts have been recognized in CO_2_‐RR for liquid carbon product selectivity. Generally, Cu catalysts have the advantage of enhancing the product selectivity to liquid fuels, and mostly, C_2_+ products over other metals. Cu/C catalysts derived from the carbonization of Cu‐HKUST‐1 at different temperatures successfully converted CO_2_ to alcohols with 45.2–71.2% FE and exhibited high activity, high selectivity, and good stability for the CO_2_RR.^[^ ^163^ ^]^ From −0.1 to −0.7 V versus RHE, the total FE for MeOH and EtOH reached 71.2%, which was attributed to the synergistic effect of highly dispersed Cu metal centers on the porous carbon supports with high stability (**Figure** **9**). These results indicate that the catalytic activity and products of Cu‐MOF‐derived catalysts are greatly related to the calcination temperatures as they directly determine the metal particle size and Cu–Carbon synergy which enhances C─C coupling. Marepally et al. defined the effects of particle sizes of Cu on carbon composite materials by depositing small Cu nanoparticles with an average size of ≈ 3 nm on carbon nanotubes (Cu NPs/CNTs) and comparing them with Cu/CNTs prepared by conventional impregnation method (Figure 9e,f).^[^ ^164^ ^]^ The obtained Cu NPs/CNTs were active in C─C coupling and more efficient in reducing CO_2_. The products contained some C_2_+ oxygenates, indicating the small size of the Cu and carbon interface can be a possible site to enhance C─C coupling. In addition, the functionalization of the carbon created oxygen functional groups acted as anchorage sites for the particles and electron traps for CO_2_ reduction.

Heteroatoms have also been incorporated in the metal‐supported carbon catalysts to enhance the electronic structure for enhanced multicarbon product formation. Han et al. investigated the influence of pyridinic N contents on carbon‐supported Cu catalysts for the multicarbon alcohol production in CO_2_RR.^[^ ^165^ ^]^ Their results concluded that the high pyridinic N content was advantageous to CO_2_ adsorption and subsequent production of CO before catalysis on the Cu/C interface. A total FE of 73.3% for C_2_H_5_OH and C_3_H_7_OH was achieved with Cu/NPC‐800. Dongare et al.^[^ ^166^ ^]^ designed oxide‐derived Cu–Zn NPs supported on N‐doped graphene (CuZn*_x_*/NGN) for CO_2_RR to ethanol. The catalyst prepared by simple co‐precipitation method followed by annealing demonstrated a high FE of 34.25% for EtOH as demosntrated in the Table 3. The NGN component of the catalysts benefitted electron conductivity and favored the mass transfer of molecules. Moreover, it limited over potential with the pyridinic‐N sites favorable for CO_2_ adsorption. Yuan et al. fabricated Cu/TiO_2_ NPs modified N‐doped graphene (Cu/TiO_2_/NG) catalyst for converting CO_2_ into different alcohols by CO_2_RR.^[^ ^167^ ^]^ The Cu/TiO_2_/NG catalyst was prepared by reduction of Cu(NO_3_)_2_ ⋅ 3H_2_O with hydrazine hydrate. During synthesis, sodium citrate was used to stabilize Cu^2+^ species. The characterization results revealed Cu in a metallic state with high pyridinic N content in Cu/TiO_2_/NG. The final catalyst demonstrated outstanding selectivity to produce ethanol with FE up to 43.6% at −0.75 V (vs RHE). Ag NPs supported on the 3D graphene‐wrapped nitrogen‐doped carbon foam (Ag‐G‐NCF) for CO_2_RR were reported.^[^ ^168^ ^]^ Ag‐G‐NCF was fabricated by carbonization of silver salt and graphene oxide loaded on melamine foam at 500 °C for 1 h; and then, at 800 °C for 1 h under an inert N_2_ atmosphere. The Ag‐G‐NCF converted CO_2_ to ethanol with the FE of 82.1–85.2% at −0.6 to −0.7 V (vs RHE). Jianzhi Huang et al. synthesized partially oxidized cobalt nanoparticles dispersed on nitrogen‐doped graphene, which could selectively electrocatalytically reduce CO_2_ to CH_3_OH with a maximum FE of 71.4% at −0.90 V (vs SHE); the electrocatalytic current density was 4 mAcm^−2^, and the corresponding overpotential was as low as 280 mV.^[^ ^169^ ^]^ Other interesting works have been done on carbon for alcohol synthesis exploiting their porosity, dispersive properties, and functional interface, aside from core traits of anchorage and conductivity as presented in the Table 3.^[^ ^170^ ^]^ The metal–carbon interface is key to CO_2_ reduction and subsequent transformation to valuable liquid products, and the addition of heteroatoms is also a confirmed path positively influencing the reaction path with enhanced CO_2_ adsorptions and C─C coupling.

**Figure 10:**
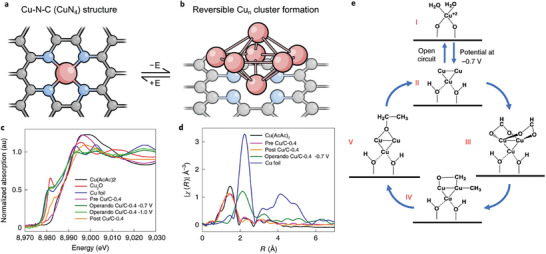
a,b) Schematic representation of the reversible formation of Cu and Cu cluster for C─C coupling over graphitic nitride support. Reproduced with permission.^[^ ^171^ ^]^ c) XANES confirming the reduction of Cu^2+^ species to Cu^O^ species. d) EXAFS showing the formed Cu─Cu in situ with reference pre‐ and post‐catalysts and e) showing the detailed cluster formation. Reproduced with permission.^[^ ^172^ ^]^ Copyright 2020, Springer Nature.

The C─C coupling step is recognized to proceed via the dimerization of two major intermediates, HCOOH* and CO*. In the area of single‐atom catalysis, the carbon supports function well in stabilizing the metal particles in the overall process. As stated by Creissen et al.^[^ ^171^ ^]^ and Xu et al.,^[^ ^172^ ^]^ the enhanced C─C coupling proceeds over a dynamic cluster of “Cu” rather than single atoms during the reaction. This reversible mechanism is achieved by carbon substrate, allowing the dynamic mechanism to proceed while keeping the particles actively small (**Figure** **10**). Zhang et al. revealed the aggregated clusters of Cu(n) as C─N─Cu_5_ phase, which enhanced the formation of ethanol via the formation of CHO species.^[^ ^173^ ^]^

## 4 Photocatalytic CO_2_ Reduction to Liquid Fuels

**Figure 11:**
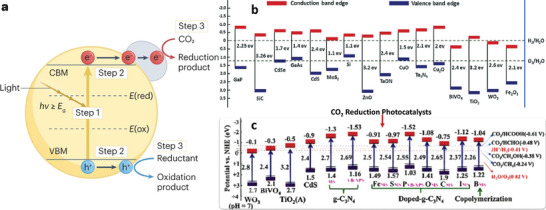
a) Simplified scheme of the photocatalytic reduction of CO_2_, b) the band gap of different semiconductor photocatalysts (against (NHE)), and c) the common CO_2_ reduction photocatalysts. Reproduced with permission.^[^ ^182^, ^183^, ^184^ ^]^ Copyright 2023, Springer Nature.

Photocatalysis defines a light‐driven reaction in the presence of a photocatalyst. A photocatalytic reaction can be viewed as the photosynthesis for green plants. Typically, this reaction can be divided into four steps: First, the catalyst is activated by the ultraviolet (UV) or visible light from sunlight or an illuminated light source such as Xenon lamps. The excited electrons jump from the valence band (VB) into the conduction band (CB) of the catalytic material leaving holes in the valence band. The generated electron–hole pairs separate and transport to the surface of catalysts where the reduction of CO_2_ and oxidation of H_2_O take place (**Figure** **11**a). Finally, the products are desorbed from the catalyst surface and related recombination of charges is done to begin the cycle. Various strategies are presented for photocatalysis, which has included the type of materials and reactions. Key influencers in CO_2_ photocatalysis are the relaxation time of the photoexcited charges and light adsorption capacity. Popular catalysts for CO_2_ reduction to various fuels are displayed in Figure 10a,b, which include some semiconductors, MXenes, perovskites, and carbon‐based materials. Carbon materials are gaining popularity with their ability to absorb light within a wider wavelength, a limitation associated with semiconductors, and thus; materials such as CNTs, graphene, carbon nitrides, and Mo_2_C have functioned catalytically within these descriptions for effective CO_2_ reduction to liquid fuels.^[^ ^179^ ^]^ A carbon decorated TiO_2_ is developed for the direct conversion of CO_2_ to CH_3_OH.^[^ ^179^ ^]^ The authors conclude that the light absorption is improved due to the black color and zero‐band gap of the carbon, which in turn, leads to the generation of electrons with high energy. Another report by Brunetti et al. decorated TiO_2_ with graphitic nitride, as in C_3_N_4_–TiO_2_, as a catalyst for enhanced CH_3_OH production.^[^ ^180^ ^]^ A similar catalyst composition was developed by Kavil et al. for CH_3_OH production.^[^ ^181^ ^]^ However this time, the inclusion of a Cu metal further improved the absorption of UV light with further suppression of electron–hole recombination. More success in the use of carbon‐based catalysts in photocatalytic CO_2_ reduction to liquid fuels is reported in **Table** **4**. Photocatalytic reactions could further be combined with thermal or electro‐ energy sources as in photothermal and photo‐electrolytic reactions.

**Table 4:** Carbon‐based photocatalyts in CO_2_ conversion to liquid fuels.

Photocatalyst	Light source	Catalyst preparation	Product.(Yield^a)^/selectivity^b)^)	Ref.
NiO*_x_*–Ta_2_O_5_–rG	500 W Xe lamp	Hydrothermal method	CH_3_OH. 20 µmol^a)^	[196]
LaYAgO_4_–graphene–TiO_2_	500 W halide lamp	Hydrothermal method	CH_3_OH. 12.27%^b)^ (1945.9 mmol^.^g_cat_^−1a)^	[197]
Cu_2_O/rGO	300 W Xe lamp	Solution‐chemistry route	CH_3_OH. 355.3 mmol^.^g^−1a)^	[198]
CuCaAg_2_Se–graphene–TiO_2_	500 W metal halide lamp	Muffle‐assisted hydrothermal method	CH_3_OH. 16.84%^b)^	[199]
STO/Cu@Ni/SiO_2_	300 W Xe lamp	Coprecipitation	CH_3_OH. 76.9 µmol^.^g^−1a)^	[200]
InCu/PCN	300 W Xe lamp	—	CH_3_OH. 28.5 µmol^−1.^g^−1^h^−1a)^	[201]
CD/CN	300 W Xe lamp	Microwave‐heating method	CH_3_OH. 99.6%^b)^	[202]
CNNA/rGO	350 W Xe lamp	Ionothermal method	C_2_H_5_OH.1.15 µmol^.^gcat^−1^.h^−1a)^	[203]
CNNA/rGO	350 W Xe lamp	Ionothermal method	CH_3_OH.0.53 µmol^.^gcat^−1^.h^−1a)^	[203]
rGO‐NH_2_‐MIL‐ 125(Ti)	20 W visible light white cold LED lamp	Hydrothermal method	CH_3_OH. 47.2 mmol^.^g^−1a)^	[204]
AgCuInS_2_–G–TiO_2_	Halide lamp (500 W)	Hydrothermal method	CH_3_OH. 15.21%^b)^	[205]

### 4.1 Photothermal CO_2_ Conversion

**Figure 12:**
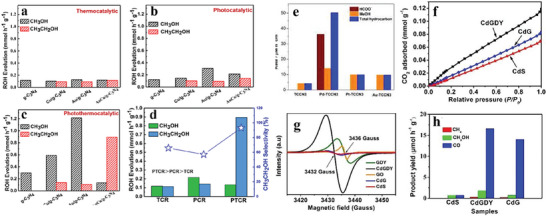
a–d) Catalyst activity and selectivity to fuels over AuCu/g‐C_3_N_4_ catalyst in CO_2_RR. TCR = thermal catalytic reactions, PCR = photocatalytic reaction, and PTCR = photo thermal catalytic reaction; e) Metal modified CN and f–h) graphdiynde modified CdS in CO_2_ hydrogenation. Reproduced with permission.^[^ ^189^ ^]^ Copyright 2020, Elsevier. Reproduced with permission.^[^ ^193^, ^195^ ^]^ Copyright 2020, Royal Chemical Society.

The photo‐thermal catalysis for CO_2_ conversions combines photo and thermal sources for CO_2_ conversion. These have been proven to increase the conversion rate of CO_2_.^[^ ^185^, ^186^ ^]^ The photothermal conversion of CO_2_ normally proceeds in a liquid medium (CO_2_+H_2_O) due to the limited efficiency revealed to follow the direct hydrogenation of CO_2_ (CO_2_+H_2_) in a photocatalytic system.^[^ ^187^, ^188^ ^]^ So far, progress in this domain is mainly focused on the synthesis of oxygenated fuels. In this respect, carbon materials are being exploited for successfully relying on their oxygenated functional groups and bonds for better light absorption and auxiliary functions. A Na–Co@C catalyst is developed to improve the selectivity to C_2_+ hydrocarbons and alcohol.^[^ ^186^ ^]^ The results indicate that more ethanol is produced at lower conversions under photothermal conditions. The carbon layer functions mainly for electron generation. Li P. et. al. investigated ultrathin porous AuCu/g‐C_3_N_4_ nanocomposite for photothermal CO_2_ conversion to ethanol.^[^ ^189^ ^]^ The photothermal activity recorded was 5.6 and 3.9 times higher compared with that of thermal catalysis and photocatalysis, respectively. In addition, the bimetallic AuCu/g–C_3_N_4_ showed a high ethanol selectivity of 93.1%, which was higher than that of the Cu/g–C_3_N_4_ and Au/g–C_3_N_4_ catalysts, as presented in **Figure** **12**. The authors iterated that the g‐C_3_N_4_ functioned for metal dispersion as well as enhancing the synergy between the metal nanoparticles. The photothermal catalysis approach successfully improved the synthesis of ethanol over solely thermal and photo‐catalyzed CO_2_ conversion using a Cu_2_O/g–C_3_N_4_ catalyst.^[^ ^190^ ^]^ The combined photothermal conditions (UV–vis + 100 °C) yielded 0.71 mmol⋅g^−1^⋅h^−1^ ethanol, which was 1.89 times and 7.05 times that of photocatalysis and thermal catalysis reactions, respectively. The graphs below show that the g‐C_3_N_4_ demonstrated no activity toward ethanol production whereas Cu_2_O showed little activity to EtOH. The high activity demonstrated by the Cu_2_O/g–C_3_N_4_ catalysts could be ascribed to the improved CO_2_ adsorption over the composite catalyst. In addition, the light absorption was improved for both Cu_2_O and g‐C_3_N_4_ at the heterojunction interface, which was recognized to improve the photocatalytic activity.

### 4.2 Photoelectrochemical CO_2_ Conversion

Aside from thermally assisted routes, photocatalysis can also be combined with electrolysis for efficient CO_2_ reduction to fuels. Combining both photo‐ and electro‐catalytic reactions results in a reduction of the overall input energy. In addition, a better separation and transmission of the charges can be achieved. The production of liquid fuels via this path has seen some applications of carbon‐based catalysts in the process. The graphitic nitride, g‐C_3_N_4,_ remains the popular carbon material for the reduction process and charge transfer, However, modifications in terms of heteroatoms and metals have proven to further increase the reduction and product formation by multiple folds.^[^ ^191^ ^]^ Sagara et al. prepared a boron‐doped g‐C_3_N_4_ (BCN*x*) electrode for photoelectrochemical reduction of CO_2_ under visible light irradiation.^[^ ^192^ ^]^The BCN*x*, in addition to changing the energy band structure, demonstrated remarkable p‐type conductivity and further enhanced with the highest selectivity for C_2_H_5_OH.^[^ ^193^ ^]^ Xu et al.^[^ ^193^ ^]^ fabricated Ti_3_C_2_/g–C_3_N_4_ (TCCN) heterojunctions for photo electrocatalytic CO_2_ reduction. The heterojunction not only enhanced light absorption but also promoted charge separation to increase the photocurrent. As shown in Figure 10, the effects of different metallic species supported on graphitic nitrides were established with Pd demonstrating high activity to formate and MeOH. A thorough investigation of the effects of carbon was reported by Kang et al. on rGO‐modified TiO_2_ cathode for EtOH production in the photoelectrochemical process.^[^ ^194^ ^]^ The reaction was conducted with varied reduction degrees of rGO on TiO_2_. The results indicate that the catalysts reduced for 3 h promoted the efficient transport of electrons from the dark cathode to the CO_2_ molecules for liquid fuel synthesis with a high FE of ≈95%. A graphdiynde modified CdS was reported to improve the photochemical conversion of CO_2_ to methanol.^[^ ^195^ ^]^ The catalyst was composed of “S” which suppressed the recombination of charges generated. In addition, the acetylenic linkage in the CdS/graphdiynde catalysts was deficient in electrons enhancing the adsorption of CO_2_.

## 5 Conclusions and Outlook

### 5.1 Conclusions

Synthesis of clean fuels is now pivotal for a low‐carbon economy. This can be attained by lowering the emissions mainly at commercial sites of applications. For this reason, research into various GTL technologies such as CO_2_RR and CO_2_ hydrogenation has increased as alternative routes for fuel synthesis. While the long‐term feasibility of this approach cannot be doubted, the concept for industrial applications will factor in catalytic processes which mainly function as the key to various targets. Currently, the renewable synthesis of some gaseous products has seen breakthroughs with some industrial applications products over conventional options. The challenge now lies in catalytic materials to orient the products toward more liquid fuels. Among materials popularly sought are carbon materials with characteristics attractive across the various platforms. Their versatility and tunable properties are advantageous for the synthesis of various liquid products.

The content of this work critically reviews the influence of carbon materials across heterogeneous CO_2_ conversion to liquid fuels. The research highlights the carbon materials participating in thermo‐, electro‐, and photocatalytic reactions, mainly focusing on their characteristics. In the thermo‐catalytic approach, carbon is identified to perform the functions of anchorage with ample SMI, allowing the metal phases of the catalyst to function without much limitations. Common attributes identified from the MOF‐derived catalysts indicate that the carbon framework strongly resists the mobility of the metal phases of the catalysts. The carbon materials also create a surface to load more active metal catalysts. Furthermore, the carbon materials can create oxygen vacancies on their surfaces for the formation of Fe_5_C_2_/e‐Fe_2_C and Co_2_C identified as key phases and also facilitated on carbon supports, making the carbon material key for oxygenate fuel synthesis. In addition, the reversible formation of Cu‐clusters, key for the C─C coupling step is facilitated over carbon supports, keeping carbonaceous materials key in the drive toward a sustainable future. Another key revelation is that graphitized carbon supports activity with high conversion and high selectivity for C_5_+ hydrocarbons. The graphitic carbon surface can further improve the dissociative adsorption of H_2_ and reduce the reduction temperature of the catalysts. To further facilitate chain‐growth, the electron‐rich exterior of carbon supports can bind to the primary hydrocarbons to further enhance the selectivity for liquid hydrocarbons. Furthermore, nitrogen is also an e functional material on carbon in CO_2_ hydrogenation reactions. The pyridinic‐N performs better for CH_3_OH selectivity and CO_2_ activation. It is also recognized to favor C─C coupling for C_2_+ product formation.

In the electrocatalytic process, carbon materials are mainly exploited for their tailorable porous structures, high surface area for adsorption, and high electrical conductivity. Normally, an intrinsic carbon material will display limited electrochemical activity; however, modifications in terms of hetero‐atom doping and metals can alter the properties for effective electrochemical CO_2_ reduction. Unlike the thermo‐catalytic approach, the pyrrolic is more active in CO_2_RR with reduced activity to HER. Photocatalysis keeps growing in the reduction of CO_2_ with carbon materials playing a key role in charge generation and transfer. The light absorption potential, large relaxation time of charges, and efficient transport of charges are making carbon materials popular in recent applications. Further modifications with heteroatoms and metals are proven to enhance the use of carbon materials for reducing CO_2_ to liquid fuels.

### 5.2 Outlook

The future of carbon materials cannot be doubted; however, commercial applications will require standardized characteristics of efficient catalysis to target products. To date, effective use of carbon material for enhanced selectivity to heavy hydrocarbons is sparse. Benchmark research confirming all necessary traits of carbons necessary for C─C coupling needs to be outlined as literature for subsequent research and applications. In the area of thermo‐catalysis, the formation of active phases is influenced by carbon materials. Investigations to regulate these active phase formations and their activity need better elucidation. In addition, other undesirable effects of the carbon materials in the reaction process, which are currently very limited, need to be addressed for optimum activity.

Looking at the use of carbon materials in electrochemistry, the advantage of low cost and facile preparation procedures makes the use of carbon attractive. However distinct function in the C─C chain growth for liquid fuels production is not so clear. Currently, CO_2_ conversion to oxygenated fuels dominates among liquid fuels with little success to long chain hydrocarbon. More research on this can be beneficial in future catalyst development. Likewise, photocatalytic CO_2_ conversion has little success in long‐chain hydrocarbon production. Although CO can be converted to long‐chain hydrocarbons with high carbon number in a photocatalytic FTS process, the reduction of CO_2_ with FTS as a subsequent step is challenging. More research exploiting carbon functionalities for reabsorption capabilities can help target these long chain hydrocarbons. What's more, more research highlighting the key carbon characteristics in the reduction process for enhanced C─C coupling is necessary.

While this work reports constant increase in the use of carbon materials in CO_2_ conversion processes, the parallel application at the industrial front is sparse. There are still major issues with catalyst regeneration popularly associated GTL processes. The oxidative treatment popularly employed may pose a challenge for carbon‐based catalysts. In addition, pelletizing carbon catalysts remains a challenge as the final granule tends to possess a weaker mechanical strength compared with other supports. These key challenges will need to be addressed in order to make carbon‐based catalysts competitive in the developing robust catalysts in CO_2_ conversion. The scope for carbon‐based catalysts is still wide with the development of new carbon structure for various catalytic applications. More research on facile preparation procedures and development of stable carbon structure and composite are still plausible.

## Conflict of Interest

The authors declare no conflict of interest.
